# Distinguishing injury patterns in fatal falls from heights versus pedestrian impacts: an autopsy study for differential diagnosis in ambiguous cases

**DOI:** 10.1007/s12024-024-00888-3

**Published:** 2024-09-13

**Authors:** Alessandro Mauro Tavone, Roberta Marinelli, Francesca Cazzato, Giorgia Piizzi, Federico Piselli, Giulia Ceccobelli, Gabriele Giuga, Raimondo Vella, Naomi Romaniello, Antonio Oliva, Gian Luca Marella

**Affiliations:** 1 Department of Surgical Sciences, University of Rome Tor Vergata, Via Montpellier 1, Rome, 00133 Italy; 2 Department of Health Surveillance and Bioethics, Section of Legal Medicine, Fondazione Policlinico A. Gemelli IRCCS, Università Cattolica del Sacro Cuore, Largo Francesco Vito 1, Rome, 00168 Italy; 3 Department of Biomedicine and Prevention, University of Rome Tor Vergata, Via Montpellier 1, Rome, 00133 Italy

**Keywords:** Differential diagnosis, Falls from height, Pedestrian, Autopsy, Injury patterns, Forensic pathology

## Abstract

This study investigated the injury patterns associated with fatal falls from heights compared to individuals struck by cars, aiming to enhance the differential diagnosis in ambiguous cases, where it is unclear whether the body fell from nearby building or was left on the street following a road traffic incident. A retrospective review of comprehensive forensic reports from the Institute of Legal Medicine of the University of Rome “Tor Vergata” between 2012 and 2023 was conducted. The analysis included 232 cases, gathering data on internal organ injuries, skeletal fractures, external skin injuries, as well as pleural, peritoneal, and pericardial effusions. Bilateral lung injuries were significantly more common in falls from height (33.3%) compared to pedestrians (13.6%, *p* < 0.001). Liver injuries also occurred more frequently in fall victims (49.6%) than in pedestrians (28.2%, *p* < 0.001). Skull fractures were more frequent in falls from height (68.2%) versus individuals struck by cars (55.3%, *p* = 0.044), while unilateral leg fractures were more common in pedestrians (28.2%) compared to fall victims (16.3%, *p* = 0.029). External injuries, notably to the head and legs, were more frequent in pedestrians. The “Total Injured Skin Area” analysis revealed a significant discriminative power with an optimal cut-off of 84.2 cm², suggesting that injuries exceeding this threshold may be indicative of a pedestrian road fatality.

## Introduction

Road traffic incidents and falls from height are common trauma-related deaths encountered by forensic pathologists during autopsies [1–4]. According to the “*Global Status Report on Road Safety 2023*” by the World Health Organization (WHO) [5], road traffic incidents result in approximately 1.19 million deaths annually, with a road fatality rate of 15 per 100,000 people. The distribution of these fatalities varies significantly among different types of road users. Pedestrians represent 21% of the global road traffic deaths, while motorcyclists account for 30%. Occupants of four-wheeled vehicles represent 25% of these fatalities, and cyclists make up 5%. The remaining 19% comprises other users, including those in vehicles carrying more than ten people, heavy goods vehicles, and various other categories. Notably, there are limited countries with comprehensive laws aimed at protecting pedestrians, cyclists, and other vulnerable road users, highlighting a critical gap in global road safety measures.

Moreover, according to the WHO [6] falls from height are a significant public health issue globally, resulting in approximately 684,000 fatalities each year, making them the second leading cause of unintentional injury death after road traffic incidents. Fatal falls from heights are frequently associated with either suicidal intent or accidental events, with contributing factors including the consumption of alcohol and the administration of psychoactive substances [7].

Both these events are commonplace in forensic pathology worldwide. Differentiating between a fall from height and an individual struck by a car, although seemingly rare, is crucial in cases where a body is discovered on the street without eyewitnesses. In such scenarios, the cause of death could be either a fall from a building or a hit-and-run accident. For diagnostic purposes, the absence of extensive external injuries or a concentration of severe internal injuries may indicate a fall [8]. However, diagnosis can be challenging, especially if the fall involved obstacles that caused injuries without halting the body’s descent, requiring thus a thorough investigation of the suspected fall site.

In instances of falls from heights, the severity and location of injuries are frequently associated with the height of the fall (e.g., medium: below 10 m; greater: above 10 m), the victim’s age and comorbidities (e.g., osteoporosis), as well as the trajectories and orientation of the body during the descent. The site of the primary impact is often where the most severe injury occurs; however, this is not always the case. The impact may simultaneously affect two body areas, or it may involve a ricochet effect, resulting in multiple major impacts in rapid succession [9]. When the body falls onto the head, massive skull vault and base fractures are likely to occur, often accompanied by lacerations of the scalp and possibly by extrusion of the brain and spinal injuries. Falls onto the feet commonly result in “ring fractures” of the skull, as well as fractures of the lower limbs and pelvic girdle. Falls on the side can cause multiple rib fractures, shoulder girdle or arm fractures, lacerations of back, buttocks or limbs and severe abdominal injuries. Internal lesions are usually represented by ruptures of the brain, liver, lungs, heart, aorta, or spleen [9].

In pedestrian road fatalities, primary injuries are typically caused by the vehicle’s initial impact, while secondary injuries result from subsequent ground contact [10]. These fatalities traditionally follow a characteristic sequence of events related to body acceleration. The first phase is represented by the impact of the vehicle with the pedestrian’s body (usually below its gravity center), which may result in projection forward or a scooping-up motion (i.e., second phase). During the third phase, the victim hits the ground in front of the car and may even be run over during the vehicle’s residual movement before it finally comes to a stop [10]. However, the movement of the body following impact may be influenced by factors such as hard braking. Additionally, the speed of the motor vehicle plays a crucial role in determining the pattern and severity of injuries. Therefore, differences in injury patterns may be observed between urban settings -where traffic conditions and speed limits generally result in lower velocities- and rural areas -where vehicles often travel at higher speeds. As a result, forward displacement of the pedestrian following impact is most observed in low-speed impacts following hard braking [11]. The most common traumas are to the legs, with abrasions, lacerations, and often compound fractures of the tibia and fibula. Skull fractures, especially of the base, are also frequent, followed by fractures of the chest, arms, pelvis, and abdominal injuries. Site investigation and autopsy findings, focusing on bruises and abrasions, are essential to determine the circumstances of the collision [10].

Differentiating between these two types of death relies heavily on both testimonial evidence and forensic findings. External examination of the body and forensic autopsy are critical in providing key insights into the injury patterns. However, determining the cause of death may be challenging in cases where there is no direct witness testimony and when the injuries are ambiguous and could be consistent with either a fall from height or an impact from a motor vehicle. Thus, as forensic evidence is presented in courtroom, it is essential to develop a reliable diagnostic method to be applied in forensic settings to differentiate between the homicide, suicide and accidental scenarios associated with these types of fatalities.

This study aims to compare injury patterns in car- pedestrian impacts and falls from heights that were subjected to forensic examination at the Institute of Legal Medicine of the University of Rome “Tor Vergata” from 2012 to 2023. The purpose is to identify significant differences that may assist forensic pathologists in differential diagnosis, ensuring an approach characterized by a high degree of objectivity essential for judicial investigations.

## Materials and methods

### Study Population, variables and data Collection

In the current study, four of the authors conducted a review of all comprehensive forensic reports -encompassing autopsy findings as well as circumstantial information, witness testimonies, and crime scene inspection details- performed at the Institute of Legal Medicine of the University of Rome “Tor Vergata” between 2012 and 2023. During a preliminary assessment, all cases were identified where the cause of death was attributed to either being struck by a car or falling from a height.

The cases were then selected based on the following inclusion and exclusion criteria.

Inclusion criteria:

- available circumstantial information and documented witness testimony of the death;
- full forensic examination of the body performed, including both external examination and autopsy;
- cause of death exclusively and definitively attributable to pedestrian impact or a fall from a height.

Exclusion criteria:

- impacts involving a dynamic other than a pedestrian being hit by a car (e.g., motorcycle, bicycle, rail vehicles, heavy vehicles > 2500 Kg);
- falls from heights greater than 20 m;
- falls from low heights (e.g., from standing, a chair, a ladder).
- survival of individuals for more than 24 h, due to potential changes in injury patterns;
- cause of death involving a combination of causes (e.g., both assault and fall from height);
- cause of death ambiguous or not unequivocally determined.

A data extraction form was created in Microsoft Excel v.2302 and two separate databases were established, one for each category of fatality.

For each case, 45 qualitative variables were assessed, categorized into three groups (A1; A2; A3) and recorded in a binomial fashion based on their presence or absence. The hypothesis that ground impacts might produce greater forces and affect a larger area of the body’s surface, contrasts with car-pedestrian collisions, which usually involve lateral and localized impacts. This prompted us to investigate whether injuries were unilateral or bilateral in cases involving paired structures [12–14].

*Qualitative variables*:

1. Internal organ injuries: brain; unilateral lung; bilateral lung; unilateral kidney; bilateral kidney; liver; spleen; thoracic aorta.
2. Skeletal fractures: Skull (both neurocranium and viscerocranium); sternum; cervical/dorsal/lumbar column; unilateral ribs; bilateral ribs; unilateral pectoral girdle; bilateral pectoral girdle; unilateral pelvic girdle; bilateral pelvic girdle; arm/forearm/hand unilateral; arm/forearm/hand bilateral; thigh/leg/foot unilateral; thigh/leg/foot bilateral.
3. External skin injuries (without distinction between abrasion, contusion, laceration): head; trunk (anterior and/or posterior); arm/forearm/hand unilateral; arm/forearm/hand bilateral; thigh/leg/foot unilateral; thigh/leg/foot bilateral.

*Quantitative variables*:

- Pleural effusion (ml);
- Peritoneal effusion (ml);
- Pericardial effusion (ml);
- Total injured skin area (cm^2^).

The effusion volumes were recorded as indicated by the pathologist in the autopsy report. All types of effusion, regardless of their nature -such as serous, serohaematic, or haematic- were considered. For pleural effusion, the total volume for each case was calculated as the sum of the effusions on both sides. In assessing the injured skin area, no distinction was made regarding the type of injuries (i.e., all abrasion, contusion, laceration, or incised wounds were equally considered).

In the autopsy reports, the shape and dimensions of the skin’s wounds were usually described. To quantify the total injured skin area in cm² for each case, we measured the surface area of each external injury. The area for each wound was calculated by multiplying its two maximum dimensions. Although this method tends to overestimate wound size, it provides a standardized and reproducible approach to measurement. For lacerations and incised wounds, the maximum dimensions of extension were measured without appositions of the edges. If these wounds were located within areas of contusion and/or abrasion, the measurement considered the largest dimensions of the entire wound complex. The total injured skin area per case was then obtained by summing the areas of all individual wounds.

### Statistics

#### Qualitative variables

Using SPSS software (version 26.0), a descriptive analysis and a comparison of frequencies of dependent qualitative variables - expressed in absolute values and percentages - were carried out for each of the two independent variables (event). To verify the statistical validity of the differences in the emerging frequencies, a 2 × 2 contingency table was constructed for each qualitative variable and a chi-square test was performed, assuming statistical significance for p-values < 0.05.

#### Quantitative variables

Through SPSS software (version 26.0) a descriptive analysis was conducted to estimate the mean, median, minimum, and maximum values of the dependent quantitative variables for each type of independent event. The Mann-Whitney U test was utilized to assess the significance of the differences observed between the two types of fatal events – “Pedestrian impact” and “Fall from height” - for each dependent variable, with statistical significance set at p-values < 0.05. To further refine the discriminative power of the identified variables that demonstrated statistically significant differences, Receiver Operating Characteristic (ROC) curve analysis was performed. This analysis aimed to evaluate the discriminative ability of the identified variables in classifying the two event types. The ROC curve plots the sensitivity/True Positive Rate (TPR) against the 1-specificity/False Positive Rate (FPR) or 1- specificity across various threshold settings. Here, the TPR represents the proportion of actual “Pedestrian impact” events correctly identified as such, while the FPR indicates the proportion of “Fall from height” events incorrectly identified as “Pedestrian impact”. To identify the most effective threshold for classification, the Youden Index was applied, aiming to maximize the sum of sensitivity and specificity by finding the threshold point on the ROC curve that offers the best balance between these two metrics. This methodological choice allowed for the mathematical determination of the optimal threshold value by locating the point on the ROC curve with the highest Youden Index, thus ensuring an ideal compromise between sensitivity and specificity. Regarding the treatment of missing data, the deletion method was chosen. This approach entails excluding samples with missing values for the variables under study from the ROC curve analysis. Although this strategy reduces the sample size, potentially impacting the statistical power of the findings, it preserves the original integrity of the dataset. This method proved to be straightforward and computationally efficient, avoiding potential biases introduced by imputation techniques, particularly suitable when missing data is minimal or completely random.

## Results

Among the 232 cases included in our study, 103 were deaths resulting from pedestrian traffic collisions, while the remaining 129 were due to falls from height. The data obtained from our analysis are detailed below.

### Qualitative variables

#### Category A1—Internal organs injuries

Table 1 shows the results emerged from the study of organ injuries. Analysis of internal organ injuries revealed statistically significant differences between fatalities resulting from pedestrian collisions and those resulting from falls from heights. Bilateral lung injuries were significantly more common in fall victims (*n* = 43; 33.3%) than in pedestrians (*n* = 14; 13.6%), with a p-value of < 0.001. Additionally, liver injuries were notably more frequent in falls from height (*n* = 64; 49.6%) compared to pedestrian impacts (*n* = 29; 28.2%), with a p-value of < 0.001. No other significant differences were observed between the two types of fatalities with regard to injuries to internal organs.

**Table 1:** Frequencies of internal organs injuries according to the cause of death

Internal organ injury	Pedestrian (103)	Fall from height (129)	*p*-value
Brain, n (%)	36 (35.0%)	45 (34.9%)	0.991
Lung - unilateral, n (%)	22 (21.4%)	20 (15.5%)	0.250
Lung - bilateral, n (%)	14 (13.6%)	43 (33.3%)	< 0.001*
Kidney - unilateral, n (%)	10 (9.7%)	14 (10.9%)	0.776
Kidney - bilateral, n (%)	2 (1.9%)	2 (1.6%)	0.820
Liver, n (%)	29 (28.2%)	64 (49.6%)	< 0.001*
Spleen, n (%)	17 (16.5%)	32 (24.8%)	0.124
Thoracic Aorta Rupture, n (%)	13 (12.6%)	28 (21.7%)	0.715

Chi-square test performed. *indicates statistical significance

#### Category A2— skeletal fractures

Table 2 shows the fractures found in the autopsy cases of study. Statistically significant differences were found in several fracture types. Skull fractures, including both neurocranium and viscerocranium, were more frequent in fall-from-height cases (*n* = 88, 68.2%) compared to pedestrians (*n* = 57, 55.3%), with a p-value of 0.044. Bilateral forearm fractures were notably higher in falls from height (*n* = 9, 7.0%) versus pedestrians (*n* = 0, 0.0%), with a p-value of 0.006. Additionally, unilateral leg fractures were significantly more common in pedestrians (*n* = 29, 28.2%) compared to fall victims (*n* = 21, 16.3%), with a p-value of 0.029.

**Table 2:** Frequencies of skeletal fractures according to the cause of death

Skeletal Fractures	Pedestrian (103)	Fall from height (129)	*P*-value
Skull, n (%)	57 (55.3%)	88 (68.2%)	0.044*
Sternum, n (%)	15 (14.6%)	18 (14.0%)	0.895
Cervical spine, n (%)	10 (9.7%)	22 (17.1%)	0.107
Thoracic spine, n (%)	23 (22.3%)	28 (21.7%)	0.909
Lumbar spine, n (%)	4 (3.9%)	12 (9.3%)	0.106
Rib - unilateral, n (%)	24 (23.3%)	40 (31.0%)	0.192
Rib - bilateral, n (%)	41 (39.8%)	61 (47.3%)	0.254
Pectoral girdle - unilateral, n (%)	5 (4.9%)	6 (4.7%)	0.942
Pectoral girdle - bilateral, n (%)	2 (1.9%)	1 (0.8%)	0.435
Pelvic girdle - unilateral, n (%)	17 (16.5%)	14 (10.9%)	0.209
Pelvic girdle - bilateral, n (%)	11 (10.7%)	17 (13.2%)	0.562
Arm - unilateral, n (%)	15 (14.6%)	15 (11.6%)	0.508
Arm - bilateral, n (%)	3 (2.9%)	3 (2.3%)	0.780
Forearm - unilateral, n (%)	11 (10.7%)	19 (14.7%)	0.361
Forearm - bilateral, n (%)	0 (0.0%)	9 (7.0%)	0.006*
Hand - unilateral, n (%)	1 (1.0%)	1 (0.8%)	0.873
Hand - bilateral, n (%)	0 (0.0%)	1 (0.8%)	0.371
Thigh - unilateral, n (%)	16 (15.5%)	26 (20.2%)	0.364
Thigh - bilateral, n (%)	3 (2.9%)	5 (3.9%)	0.689
Leg - unilateral, n (%)	29 (28.2%)	21 (16.3%)	0.029*
Leg - bilateral, n (%)	12 (11.7%)	11 (8.5%)	0.429
Foot - unilateral, n (%)	3 (2.9%)	9 (7.0%)	0.165
Foot - bilateral, n (%)	0 (0.0%)	1 (0.8%)	0.371

Chi-square test performed. *indicates statistical significance

#### Category A3—External injuries

The analysis of external injuries (Table 3) revealed significant associations between certain injury locations and the cause of death. Injuries to the head (87.4% vs. 75.2%, *p* = 0.020), bilateral arm (12.6% vs. 3.9%, *p* = 0.013), unilateral hand (31.1% vs. 17.8%, *p* = 0.018), bilateral thigh (19.4% vs. 9.3%, *p* = 0.026), and bilateral leg (45.6% vs. 20.9%, *p* = 0.0005) were more frequent in pedestrians. Additionally, unilateral foot injuries were also significantly more common in pedestrian impacts (19.4% vs. 7.8%, *p* = 0.009). Other injury types do not show significant differences between pedestrians and fall victims.

**Table 3:** Frequencies of anatomical distribution of external injuries according to the cause of death

External Injury	Pedestrian (103)	Fall from Height (129)	*p*-value
Head	90 (87.4%)	97 (75.2%)	0.020*
Trunk (Anterior + Posterior)	63 (61.2%)	65 (50.4%)	0.101
Arm - Unilateral	29 (28.2%)	26 (20.2%)	0.155
Arm – Bilateral	13 (12.6%)	5 (3.9%)	0.013*
Forearm - Unilateral	41 (39.8%)	36 (27.9%)	0.056
Forearm - Bilateral	15 (14.6%)	20 (15.5%)	0.842
Hand - Unilateral	32 (31.1%)	23 (17.8%)	0.018*
Hand - Bilateral	15 (14.6%)	9 (7.0%)	0.059
Thigh - Unilateral	37 (35.9%)	38 (29.5%)	0.296
Thigh - Bilateral	20 (19.4%)	12 (9.3%)	0.026*
Leg - Unilateral	29 (28.2%)	36 (27.9%)	0.967
Leg – Bilateral	47 (45.6%)	27 (20.9%)	0.0005*
Foot - Unilateral	20 (19.4%)	10 (7.8%)	0.009*
Foot – Bilateral	5 (4.9%)	4 (3.1%)	0.472

Chi-square test performed. *indicates statistical significance

### Quantitative variables

Descriptive statistics of quantitative variables are reported in Table 4.

**Table 4:** Descriptive statistic and differences in quantitative variables according to the cause of death

Quantitative Variable	Event^a^	Median	Min	Max	U-Test value	*p*-value
Pleural Effusion (ml)	Pedestrian (66)	450.0	20.0	3000.0	2857	0.529
	Fall from Height (92)	450.0	20.0	2000.0		
Peritoneal Effusion (ml)	Pedestrian (22)	50.0	40.0	1000.0	552	0.412
	Fall from Height (57)	100.0	30.0	1500.0		
Pericardial Effusion (ml)	Pedestrian (4)	50.0	40.0	200.0	22.5	1.000
	Fall from Height (11)	20.0	30.0	650.0		
Total injured skin area (cm^2^)	Pedestrian (79)	211.20	15.60	2637.7	6277.0	< 0.001*
	Fall from Height (107)	65.00	3.6	859.2		

^**a**^For effusions, the number in parentheses next to the cause of death indicates the number of cases in which the quantity of the effusion was sufficient to be measured. The minimum measured and considered is 20 ml. For the injured skin area, the number in parentheses next to the cause of death indicates the number of cases in which during the autopsy the pathologist performed a complete measurement of all external wounds. U-Mann-Whitney test performed. * indicates statistical significance

**Fig. 1:**
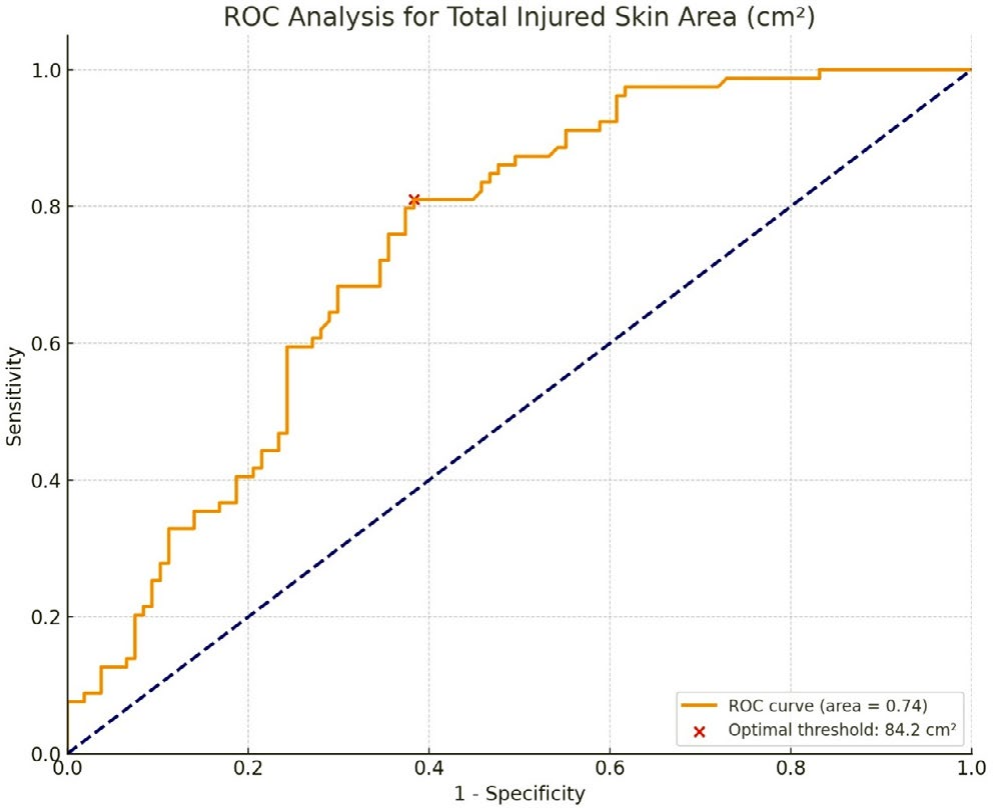
ROC Curve of total injured skin area. Red cross: This point on the curve marks the optimal cut-off value, which maximizes the difference between the rate of correct and incorrect classifications representing the coordinates of the optimal threshold value. Optimal Threshold coordinates: X-axis (sensitivity): 0.810127; Y-axis (1-Specificity): 0.383178. Optimal Youden Index: 0.426949

The Mann-Whitney U test revealed that only the “Total Injured Skin Area (cm²)” had statistically significant differences between the two types of fatal events (p-value < 0.05), warranting further discriminative analysis. Given the statistical significance of the “Total Injured Skin Area (cm²)”, Receiver Operating Characteristic (ROC) curve analysis was performed to refine its discriminative power between the two events. The analysis yielded to an Area Under the Curve (AUC) values of 0.74, indicating a good discriminative power of the variable. Figure 1 shows the ROC curve with optimal threshold coordinates.

Applying the Youden index the optimal cut-off value for classifying the event based on “Total Injured Skin Area (cm²)” was found to be 84.2 cm², with a sensitivity of 81% and a specificity of 62%.

Thus, the results of this study suggest that a threshold of 84.2 cm² may be used as a discriminative criterion in the differential diagnosis of these fatal events. Specifically, values above this threshold may be indicative of car-pedestrian collisions, whereas values below this threshold may be indicative of falls from height.

## Discussion

The differential diagnosis between injury patterns resulting from falls from heights and car-pedestrian collisions is crucial, especially in cases where the death was unwitnessed, and the body was not discovered immediately. This scenario is more common in urban environments with a high density of elevated structures, such as buildings, pedestrian overpasses, and bridges.

Our research identified several injury patterns that are significantly associated with either pedestrian or fall victims, as detailed in Tables 1, 2, 3 and 4.

The comprehensive review of forensic reports for 232 cases revealed that, in the analysis of internal organ injuries, statistically significant differences were observed only in the frequency of bilateral lung injuries and liver (*p* < 0.001). In our study, brain injuries were sustained by approximately 35% of both pedestrians (36 out of 103) and individuals who fell from heights (45 out of 129). Other studies reported that brain injuries are more common in pedestrian impacts, with prevalence rates reaching up to 94.4% when brainstem injuries are included [15–17]. We found also that bilateral lung injuries were significantly more common in falls from height (33.3%) than in pedestrians (13.6%). However, there is considerable variability in the frequency of lung involvement reported in scientific Literature of both these fatalities [13, 18–20].

In our study, the prevalence rates for unilateral kidney injuries were 9.7% in pedestrians and 10.9% in falls from heights, with no statistically significant difference between the two groups. Moreover, bilateral kidney involvement was observed in 1.9% of pedestrians and 1.6% of fall victims and was not a reliable indicator for determining the cause of death. Bansal et al. [18], reported no renal injuries among 40 fatal falls from height, suggesting the organs’ protection due to their location and size.

We also found that liver injuries were significantly more frequent in falls from height (49.6%) compared to pedestrians (28.2%). From a practical perspective and based on direct experience, it is well-established that falls from heights pose a significant risk of injury due to deceleration forces, which usually are considerably greater than those encountered in pedestrian impacts. However, these observations are variable, as they depend on factors such as the speed of vehicles and the height of the fall. Consequently, a higher frequency of organ injuries can be anticipated in falls from heights, especially affecting anatomical structures involved in organ fixation, such as the hilar regions. These injuries often present as characteristic tearing wounds in the perihilar area, involving major vessels and surrounding structures. Therefore, the higher prevalence of bilateral lung injuries and liver injuries observed in our study of falls from heights is consistent with these expectations.

Analysis of injury laterality is also crucial. Generally, pedestrians tend to experience lateral trauma upon initial impact. In contrast, falls from height are more likely to result in impacts on the frontal plane, with the head being the most common site of first impact. This difference in impact dynamics may explain the statistical variation observed in the analysis of bilateral lung injuries [21–23].

Concerning liver involvement, the observations from our case analysis align with expectations based on the aforementioned discussion. Direct impacts typically result in lacerations of the hepatic parenchyma, which are more common in pedestrians hit by cars. On the other hand, falls from heights often involve injuries to the hilar structures and perihilar parenchyma. Thus, a detailed approach focusing on the localization and qualitative differentiation of injuries in parenchymal organs, especially those associated with hilum or other fixation mechanisms, could significantly enhance the differential diagnosis process and warrants further investigation.

The analysis of skeletal fracture distribution in our cohort revealed significant differences in the prevalence of skull fractures. These fractures were commonly observed in both types of fatal events but were more frequent in falls from heights (p-value = 0.044). However, skull fractures are significantly influenced by the specific dynamics of the traumatic event. Due to the relatively small sample size, the observed differences might be due to random variation rather than indicating a true statistical difference. Further research into the spatial distribution of fractures, which was not addressed in this study, could yield additional insights.

Also, bilateral forearm fractures were notably higher in fall victims (7.0%) versus pedestrians (0.0%). On the other hand, unilateral leg fractures were significantly more common in pedestrians (28.2%) compared to falls from height (16.3%). Thus, the findings reaffirmed the validity of earlier observations regarding the laterality of fractures. Our research revealed that bilateral forearm fractures are exclusively associated with falls from heights. This finding supports the hypothesis that falls are associated with a higher frequency of frontal impacts, which may indicate a defensive posture adopted by the victims before hitting the ground.

Then, the statistical significance of the differences observed in unilateral leg fractures, which are more prevalent among pedestrians, underscores the likelihood of a lateral direct impact to the legs as a distinguishing factor in these incidents.

The literature generally does not differentiate between unilateral and bilateral lower limb injuries, often referring to them collectively as lower limb fractures. Research on vehicle-pedestrian collisions shows lower limbs involvement rates ranging from 24.2 to 52.5% [16, 24, 25]. In contrast, studies on falls from heights reveal lower rates of lower limb fractures, ranging from 6.3 to 29.8% [23, 26, 27]. Moreover, Teresinski et al. [28] reported a 30.5% incidence of foot bone fractures in individuals struck by vehicles, a rate significantly higher than the 2.9% observed in our study, with a complete absence of bilateral foot involvement.

Based on our earlier discussion of lung injuries, we expected to find differences in the frequency of bilateral rib fractures. However, such differences were not observed. This lack of disparity may be attributed to the fact that while bilateral lung injuries often result from significant deceleration forces, rib fractures are primarily caused by direct blunt trauma. Since direct blunt impact is a common factor in both falls from heights and car-pedestrian collisions, this explains the absence of significant differences in bilateral rib fractures between the two types of events, despite their higher frequency in falls from height (47.3% vs. 39.8%). However, it should be noted that the number of rib fractures was not considered in the current study, which could potentially provide additional insights for differential diagnosis.

A further aim of this study was the quantitative evaluation of specific variables, a novel approach that facilitates comparison, classification, and statistical analysis. Among the quantitative variables assessed, including pleural effusion, peritoneal effusion, pericardial effusion, and total skin injury, only the latter demonstrated statistically significant differences between deaths resulting from vehicle impacts and those from falls. The results indicated a significantly greater total injured skin surface area in pedestrians struck by cars compared to those who had fallen from heights. This finding reinforces the substantial disparity in external injuries between the two types of events.

In the current study, the “Total Injured Skin Area” threshold value of 84.2 cm², obtained through ROC analysis, represented the optimal cutoff for differentiating the cause of death. This figure might seem particularly low, especially considering that the maximum injury extent measured among pedestrians was 2637.7 cm², but it actually underscores the limited extent of external injuries recorded in cases of death due to falls from height. Among fall victims, moreover, the median total injured skin was 65.00 cm², against a median of 211.20 cm² for pedestrians. Therefore, our research suggests that injuries exceeding this threshold are likely indicative of pedestrian impacts.

However, further studies are needed to explore variables not analyzed in this preliminary research, such as fall height, impact with objects during descent, and vehicle speed. Future research could lead to the development of a diagnostic algorithm for forensic pathologists, providing a more reliable tool to differentiate between these two types of fatal events. Additionally, this study’s focus on both qualitative and quantitative data, including the patten of injuries and their mono- and bilaterality, offers promising insights that could enhance differential diagnosis. Expanding the sample size and refining the “Total Injured Skin Area” cutoff values will be crucial for integrating these findings into a comprehensive diagnostic tool.

## Limitations

The study has inherent limitations that should be considered when interpreting the data. The authors acknowledge that autopsy findings alone are insufficient to differentiate between falls from heights and car-pedestrian collisions. It is well established that determining the cause and manner of death requires comprehensive information, including the circumstances of the event, witness testimonies, and crime scene inspection details. In the absence of such information, pathologists should refrain from making a definitive diagnosis regarding the cause of death. Thus, the purpose of the current study is to assist forensic pathologists by identifying potential diagnostic markers to be applied in forensic settings. Additionally, the study’s retrospective nature and the natural subjectivity in the reporting of findings by different pathologists may have introduced bias in data collection. Other limitations may relate to the data collection methods that were chosen to ensure easy reproducibility. For instance, the study did not distinguish between different types of skull fractures, did not quantify the number of rib fractures, nor did it account for joint involvement in limbs fractures. Internal organ injuries do not differentiate wound localization into the parenchyma or at a perihilar level. External injuries do not distinguish between different types of blunt force injuries, and their measurement was not always performed by the pathologists, resulting in missing data for analysis. Moreover, some limitations are inherent and beyond control. For example, the unknown speed of vehicles can influence the energy of impact and the resulting injury patterns. Similarly, in falls from height, both the injury pattern and severity are influenced by the way the victim contacts the ground, a detail that is often unknown to the authors.

## Key points

1. The study aims to distinguish injury patterns between fatalities from falls from heights and pedestrian impacts, assisting forensic pathologists in determining the cause of death in cases without eyewitnesses.
2. The analysis includes 232 cases gathering data on internal organ injuries, skeletal fractures, external skin injuries, as well as pleural, peritoneal, and pericardial effusions.
3. Falls from height are associated more frequently with skull fractures, bilateral lung injuries, liver injuries, whereas pedestrian impacts typically present external injuries to the head and legs, as well as unilateral leg fractures.
4. A “Total Injured Skin Area” over 84.2 cm^2^ may indicate a pedestrian road fatality.

## Data Availability

The datasets generated and analyzed during the current study are available from the corresponding author on reasonable request.
